# Adult Wilms tumor revealed after radical nephrectomy for a renal mass initially suspected to be clear-cell renal cell carcinoma: a case report

**DOI:** 10.3389/fsurg.2026.1889237

**Published:** 2026-09-03

**Authors:** Kunxiang Wang, Jin Long, Cheng Pan, Yaowen Zhang, Peng Ren, Xiaojun Wan

**Affiliations:** Department of Urology, The Second Affiliated Hospital of Guizhou Medical University, Kaili, Guizhou, China

**Keywords:** adult Wilms tumor, case report, nephrology, oncology, radical nephrectomy

## Abstract

**Introduction:**

Nephroblastoma is the most common malignant renal neoplasm in children but remains exceptionally rare in adults. Compared with pediatric cases, adult Wilms tumor (AWT) often presents greater diagnostic challenges and is associated with larger tumor volumes and more advanced disease at diagnosis, contributing to poorer prognoses. Consequently, adults with Wilms tumor have significantly worse outcomes than pediatric patients. Because of the limited number of reported cases, no standardized management algorithm for adults has been established; current treatment is largely based on pediatric guidelines. To date, no optimal, universally accepted therapeutic strategy has been established, and further studies with long-term follow-up are needed.

**Case report:**

We report the case of an adult patient who was preoperatively suspected of having clear cell renal cell carcinoma and subsequently underwent a radical nephrectomy. However, postoperative histopathological examination confirmed the diagnosis of nephroblastoma. The patient subsequently received multimodal treatment according to the Children's Oncology Group (COG) EE-4A regimen. At 1 year after completion of treatment, the patient was asymptomatic and had a good performance status. Contrast-enhanced computed tomography of the chest, abdomen, and pelvis showed no evidence of residual or recurrent disease, suggesting a favorable treatment response.

**Conclusion:**

This report provides a comprehensive overview of the epidemiology, diagnostic approaches, therapeutic strategies, and prognostic determinants of adult Wilms tumor (AWT), aiming to enhance clinicians’ awareness of and ability to manage this exceptionally rare malignancy.

## Introduction

Wilms tumor, also known as nephroblastoma, is the most common malignant renal tumor in children, accounting for more than 90% of pediatric renal malignancies. However, it is exceedingly rare in adults, accounting for fewer than 0.5% of adult renal malignancies (1). Adult Wilms tumor (AWT) is conventionally defined as nephroblastoma arising in individuals aged >15 years. Its incidence is approximately 0.2 cases per million population, and fewer than 300 cases have been reported worldwide to date (2, 3). Because of its extreme rarity, AWT remains challenging to diagnose and manage. Currently, there are no adult-specific treatment guidelines, and clinical practice largely relies on pediatric Wilms tumor protocols (4).

Compared with pediatric patients, adults with Wilms tumor present greater diagnostic challenges because more common renal neoplasms, particularly renal cell carcinoma, must be considered and excluded. Consequently, adult Wilms tumor is often diagnosed at a larger tumor size and at a more advanced stage, resulting in poorer survival outcomes (5, 6). In this report, we describe the diagnostic and therapeutic course of a 39-year-old man with Wilms tumor. In addition, we review recent literature on the epidemiology, diagnostic approaches, treatment strategies, and prognostic factors associated with adult Wilms tumor to enhance clinicians' awareness of and ability to manage this rare disease.

## Case report

A 39-year-old man presented to the Department of Urology with a two-week history of persistent, progressively worsening right lower quadrant pain. He described the pain as dull, nonradiating, and unrelated to body position or food intake. He denied associated symptoms, including weight loss, fatigue, fever, night sweats, gross hematuria, nausea, and vomiting. His medical history was unremarkable, with no history of chronic conditions, including hypertension or diabetes, renal disease, surgery, or trauma. His personal and family histories were unremarkable; specifically, there was no known genetic syndrome or family history of malignancy. On physical examination, his vital signs were stable, and he was alert and oriented. The abdomen was soft, with mild deep tenderness in the right lower quadrant. No palpable mass, rebound tenderness, or guarding was noted. There was no costovertebral angle tenderness bilaterally. The remainder of the physical examination was unremarkable.

Preoperative laboratory results were unremarkable. Complete blood count values, including the white blood cell count, hemoglobin level, and platelet count, were within their respective reference ranges. Renal function tests showed normal serum creatinine and blood urea nitrogen levels. Liver function test results, including aspartate aminotransferase (AST), alanine aminotransferase (ALT), and alkaline phosphatase (ALP), were also within normal limits. Serum electrolyte levels, including sodium, potassium, and calcium, were likewise normal. Urinalysis was negative for hematuria and proteinuria.

**Figure 1:**
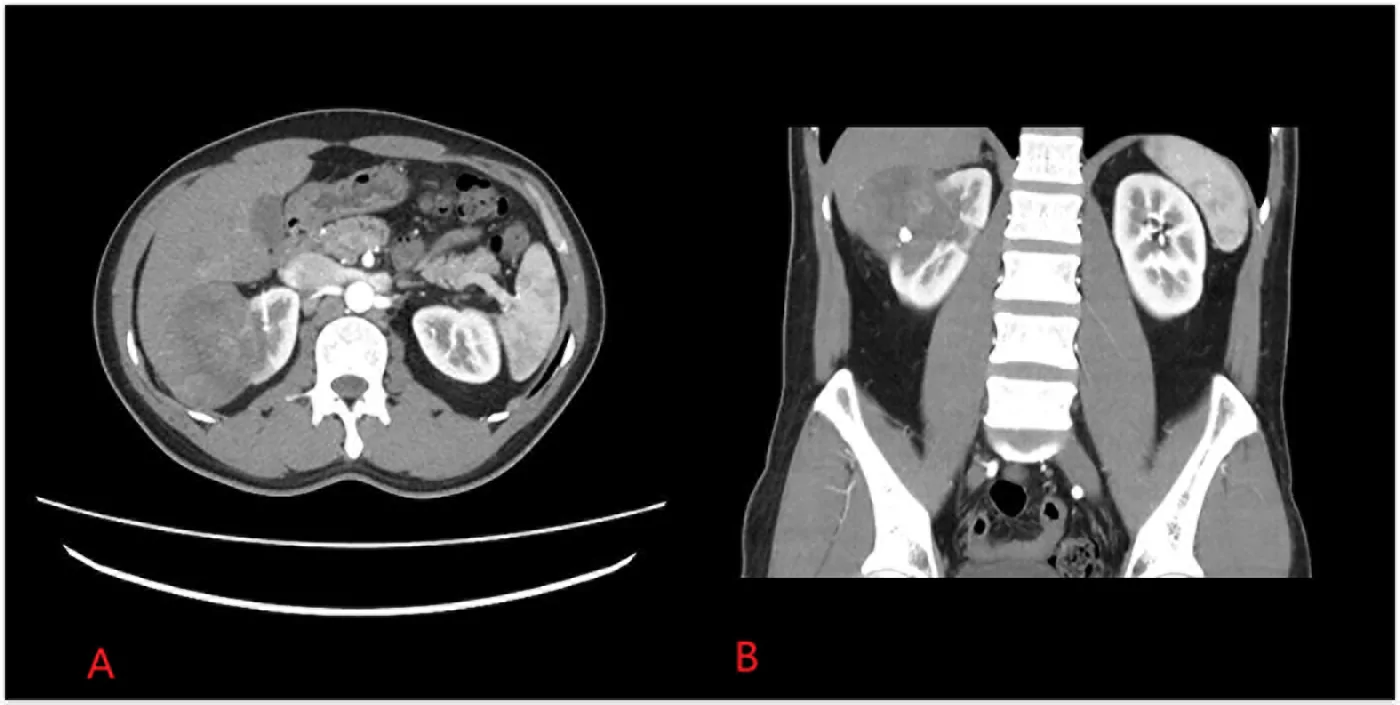
Abdominal computed tomography (CT) scan. Coronal **(A)** and axial **(B)** CT projections showed a large inhomogeneous mass measuring 72 × 65 × 66 mm in the right kidney tumor, showing a mixed density shadow, accompanied by nodular high-density lesions and patchy low-density areas, with clear boundaries, and delayed inhomogeneous enhancement after enhancement.

The patient underwent chest radiography and contrast-enhanced CT of the abdomen and pelvis. Imaging revealed a large, heterogeneous mass in the upper pole of the right kidney, measuring approximately 72 × 65 × 66 mm. The lesion demonstrated heterogeneous attenuation, with nodular hyperattenuating foci and patchy mildly hypoattenuating areas. It had well-defined margins and showed heterogeneous delayed enhancement after contrast administration. No pathologically enlarged retroperitoneal lymph nodes or distant metastases were identified (Figure 1).

**Figure 2:**
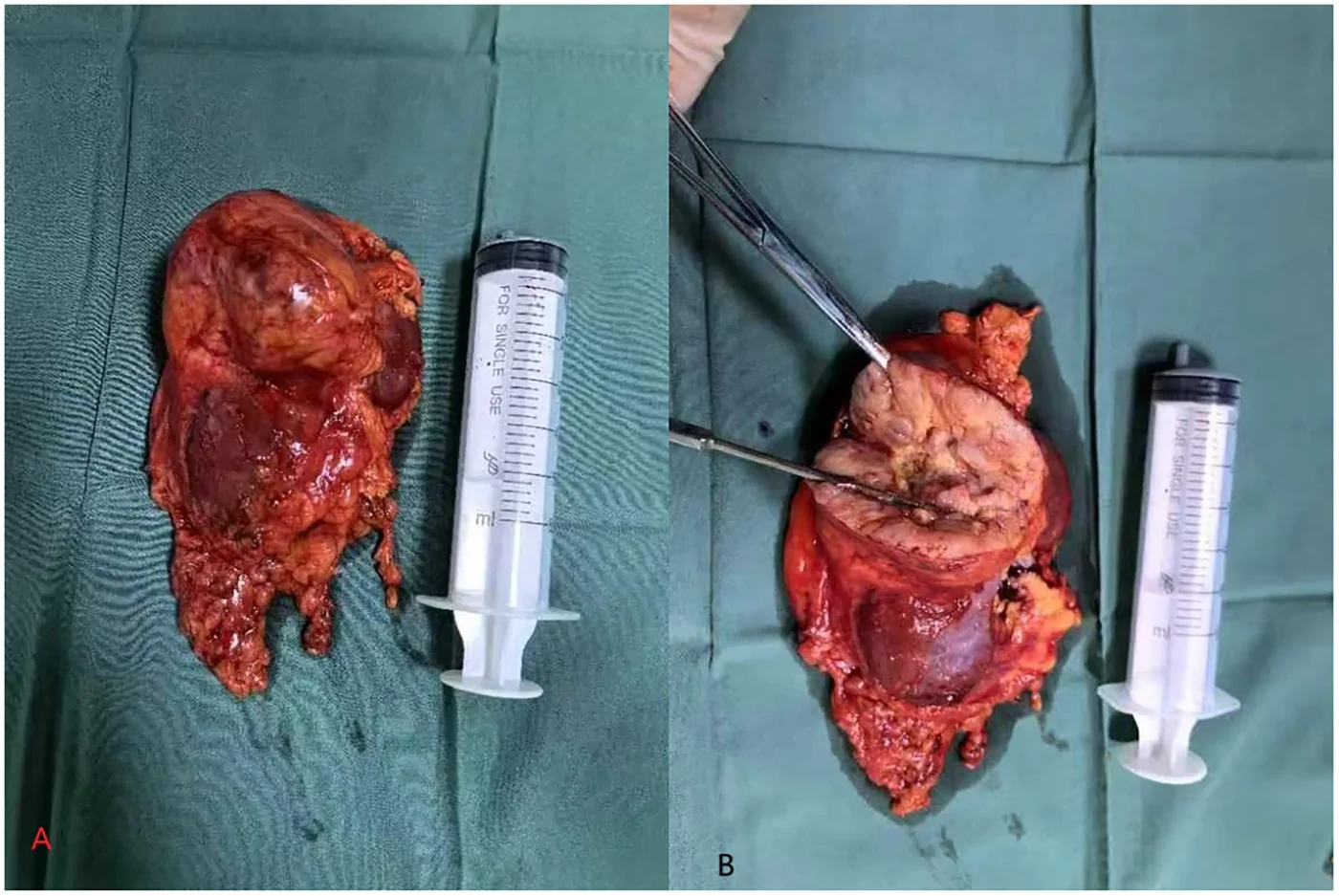
Surgical specimens after radical resection of right renal cell carcinoma showed resected tumors and affected tissues. There is a solid gray-white to gray-brown solid tumor in the upper pole of the kidney, with a size of about 7 × 6 × 6 cm, which has infiltrated the renal parenchyma and the renal pelvis/calyx system. The capsule was not ruptured, and there was no residual tumor at the resection margin and around.

**Figure 3:**
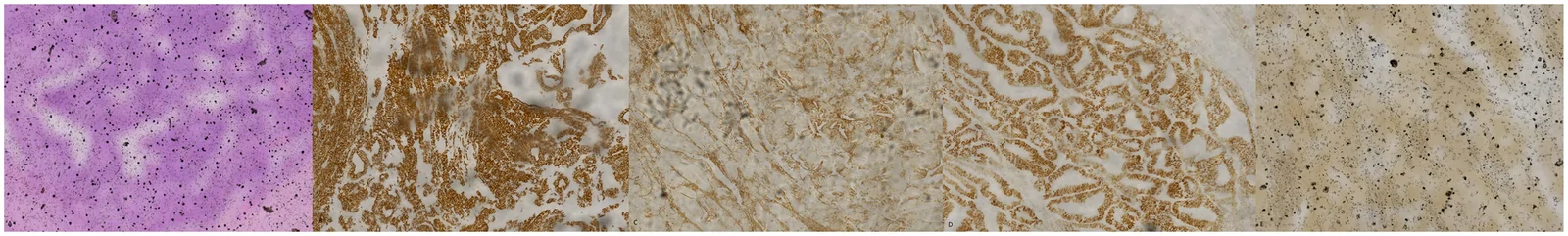
Histomorphological and immunohistochemical features of the tumor (×10). From left to right, the panels show: hematoxylin and eosin staining, demonstrating tumor cells arranged in irregular cords, trabeculae, and primitive tubules/rosette-like structures, with scant cytoplasm, hyperchromatic nuclei, and a high nuclear-to-cytoplasmic ratio; immunohistochemical staining for CK; immunohistochemical staining for Vimentin; immunohistochemical staining for PAX8; and immunohistochemical staining for WT1.

The patient underwent retroperitoneal laparoscopic radical nephrectomy. After the right renal artery and vein were clearly identified, they were sequentially ligated and divided. The right kidney, including the tumor and perirenal lymph node–bearing tissue, was removed *en bloc* (Figure 2). The procedure was completed without complications. Gross examination revealed a firm, solid, gray-white to gray-brown tumor measuring approximately 7 × 6 × 6 cm in the upper pole of the kidney. The tumor involved the renal parenchyma and renal pelvis–calyceal system. The renal capsule was intact, and all surgical resection margins were negative for tumor. Histologic examination showed a predominantly primitive epithelial tumor composed of tightly packed small- to medium-sized cells arranged in irregular cords, trabeculae, and primitive tubular or rosette-like structures. The cells had scant cytoplasm, hyperchromatic nuclei, and a high nuclear-to-cytoplasmic ratio. Brisk mitotic activity was observed, with a Ki-67 labeling index of approximately 60%. In focal areas, irregular tubules lined by cuboidal to low-columnar primitive epithelial cells supported epithelial differentiation. The morphologic and immunohistochemical findings supported nephroblastoma (Wilms tumor) with predominant epithelial differentiation (Figure 3). The tumor cells were positive for WT-1, cytokeratin, vimentin, PAX-8, and CD117, and negative for CK7, CD10, P504S/AMACR, and inhibin. The absence of clear-cell morphology, papillary architecture, CD10 expression, and P504S/AMACR expression made clear cell and papillary renal cell carcinoma less likely. The tumor was classified as stage I according to the stated Wilms tumor staging system, provided that all relevant staging criteria were met.

After multidisciplinary evaluation by urology, oncology, and radiology, the patient was classified as having stage I disease. Risk stratification was determined according to the applicable COG protocol, taking into account histology, stage, age, tumor weight, lymph node status, metastatic status, and completeness of surgical excision. No cytogenetic or molecular testing was performed to assess adverse prognostic biomarkers, including chromosome 1q gain and loss of heterozygosity (LOH) at 1p and 16q. Four weeks after surgery, the patient began treatment with the COG EE-4A regimen, which consisted of vincristine (1.5 mg/m^2^; maximum dose, 2 mg) and dactinomycin (0.5 mg/m^2^), both administered intravenously on day 1 of each 21-day cycle. The regimen was administered for six cycles at 21-day intervals. During treatment, the patient underwent serial complete blood counts, liver and renal function tests, imaging studies, and clinical evaluations to monitor treatment-related toxicity and assess for residual or recurrent disease.

**Figure 4:**
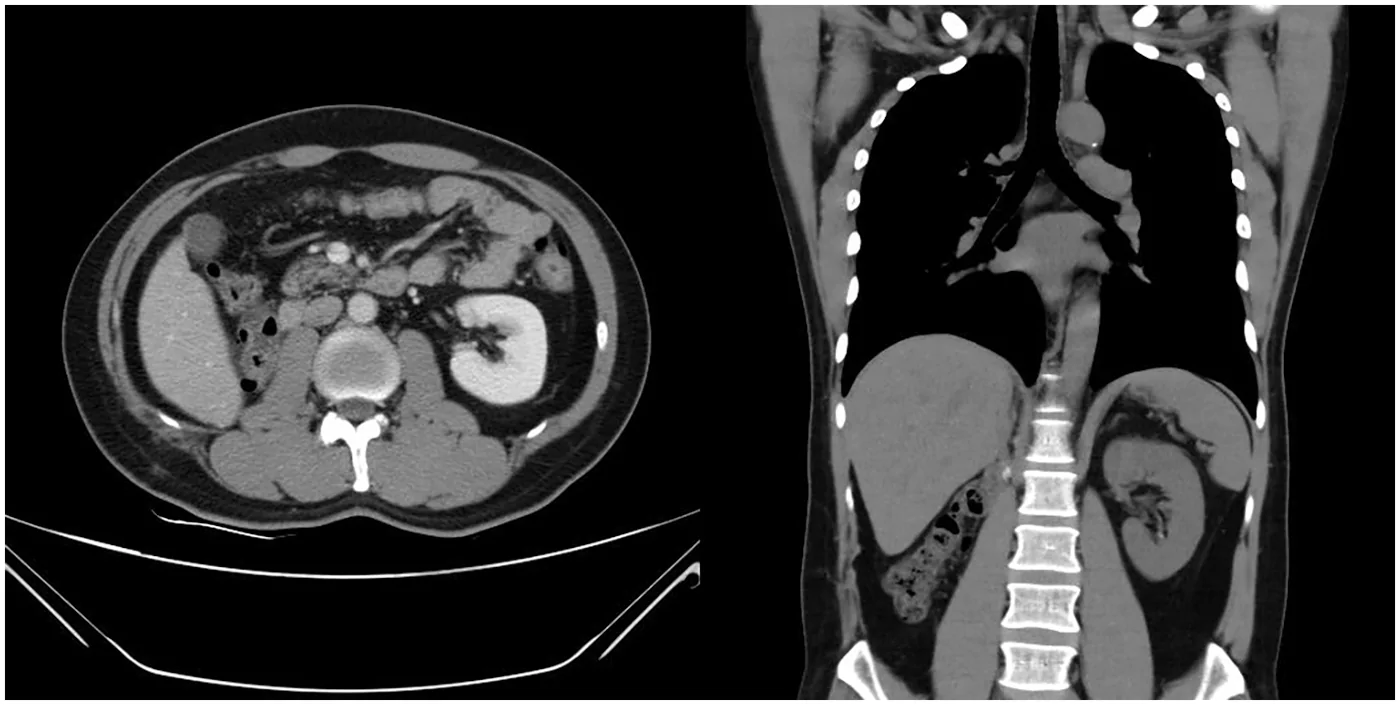
A follow-up enhanced CT scan of the chest, abdomen, and pelvis one year after comprehensive postoperative treatment showed no active tumors.

At 1 year after completing multimodal treatment, the patient remained asymptomatic and free of recurrence, with an excellent performance status. He tolerated surgery and adjuvant EE-4A chemotherapy well, with no documented treatment-related adverse events or persistent symptoms. His quality of life was maintained, and he was able to resume normal daily activities. Contrast-enhanced CT of the chest, abdomen, and pelvis showed no evidence of residual, recurrent, or metastatic disease (Figure 4). Given the risk of late relapse in adult Wilms tumor, long-term surveillance was planned to monitor for recurrence and treatment-related sequelae.

Written informed consent was obtained from the patient for publication of this case report and the accompanying images.

## Discussion

Wilms tumor was first described by the German surgeon Max Wilms in 1899. Although Wilms tumor accounts for approximately 5% of all childhood cancers and occurs in approximately 1 in 10,000 children, adult-onset Wilms tumor remains extremely rare (7).

The diagnosis of adult Wilms tumor (AWT) requires integration of clinical features, imaging findings, and histopathological confirmation. Unlike pediatric Wilms tumor, which often has more recognizable clinical and radiologic features, AWT usually presents with nonspecific and variable manifestations that can mimic other adult renal neoplasms, particularly renal cell carcinoma (RCC). Symptoms such as a flank mass, hematuria, abdominal pain, and hypertension are not specific for AWT. Similarly, CT and MRI usually show a heterogeneous renal mass, sometimes with necrosis or calcification, findings that may overlap with RCC. This clinicoradiologic overlap can create diagnostic and therapeutic uncertainty and may lead to delayed or incorrect diagnosis, potentially affecting clinical outcomes (8, 9). In the present case, the patient presented with localized abdominal pain but did not have the classic triad of abdominal pain, a palpable mass, and hematuria; hypertension and signs of metastatic disease were also absent. These features highlight the occult and heterogeneous clinical presentation of adult Wilms tumor. Consistent with many published cases, the tumor in our patient was diagnosed only after nephrectomy, which had been performed for a presumed diagnosis of renal cell carcinoma. However, unlike many previously reported adult cases, the tumor in this patient was low stage and associated with favorable prognostic features (9, 10). In adults with a heterogeneous renal mass, renal cell carcinoma (RCC), including clear cell, papillary, and sarcomatoid variants, should be the primary consideration. Other differential diagnoses include urothelial carcinoma, oncocytoma, metanephric adenoma, and primary renal primitive neuroectodermal tumor/Ewing sarcoma (PNET/ES). In this case, CT showed a relatively well-circumscribed heterogeneous mass in the upper pole of the right kidney, with delayed heterogeneous enhancement and no radiologic evidence of nodal or distant metastasis. Although useful for staging, these features were nonspecific and could not reliably distinguish Wilms tumor from other renal neoplasms. Metanephric adenoma is generally smaller and less avidly enhancing, whereas PNET/ES and sarcomatoid RCC are more often associated with necrosis, hemorrhage, infiltrative growth, venous invasion, or metastasis; however, substantial imaging overlap remains. Therefore, a preoperative diagnosis of adult Wilms tumor should be made with caution. When imaging is inconclusive and histologic confirmation would alter management, percutaneous biopsy may be considered after multidisciplinary discussion, whereas the definitive diagnosis should be based on morphology, immunohistochemistry, and, when necessary, molecular testing.

The definitive diagnosis of Wilms tumor ultimately relies on postoperative histopathologic examination (3, 6, 8). Macroscopically, Wilms tumor typically appears as a solitary, solid, well-circumscribed mass with a moist, gray-white, fish-flesh cut surface, often accompanied by hemorrhage, necrosis, and cystic change. The classic triphasic histology, comprising blastemal, epithelial, and stromal components, can be identified on routine histology and further supported by an immunohistochemical panel including WT-1, vimentin, cytokeratin, and other relevant markers. This approach helps distinguish Wilms tumor from other renal neoplasms (11). A major diagnostic challenge arises from the rarity of adult Wilms tumor, which may limit pathologists' experience; moreover, its histologic features can overlap with those of other malignant renal tumors, requiring ancillary techniques, particularly immunohistochemistry, for differential diagnosis. Accurate classification therefore requires supplementary studies, particularly immunohistochemical profiling. Nuclear WT-1 expression is present in approximately 80% of cases and is relatively specific; PAX-8 is expressed in about 70% of cases; cytokeratin is typically focally positive; and vimentin is diffusely positive. CD10 is usually negative or only weakly positive in Wilms tumor, whereas most renal cell carcinomas show strong and diffuse CD10 expression, a feature that may aid in differential diagnosis (12). In the present case, the combination of a circumscribed heterogeneous renal mass, primitive epithelial morphology with rosette-like tubular structures, diffuse WT-1 expression, and the absence of an immunophenotypic profile supporting RCC or neuroectodermal differentiation favored epithelial-predominant Wilms tumor.

The retroperitoneal laparoscopic nephrectomy with limited lymph node sampling performed in this case differs from the open transabdominal approach with systematic regional lymph node sampling recommended in pediatric Wilms tumor protocols, in which avoidance of tumor spill and accurate nodal staging are critical. However, because the preoperative diagnosis in this adult patient was renal cell carcinoma, for which laparoscopic nephrectomy is an established treatment option, the minimally invasive approach was reasonable. The procedure was completed without tumor rupture and achieved negative surgical margins, with favorable short-term postoperative control. Nevertheless, limited nodal sampling may result in understaging because occult nodal metastases cannot be excluded. Thus, laparoscopic nephrectomy should not be considered oncologically equivalent to open nephrectomy when adult Wilms tumor is suspected. In such cases, open surgery with systematic regional lymph node sampling is generally preferred. If the diagnosis is established only after laparoscopic nephrectomy, multidisciplinary review is warranted to determine the need for adjuvant therapy and long-term surveillance.

To date, high-quality evidence and adult-specific international treatment guidelines for adult Wilms tumor remain limited; therefore, treatment is generally adapted from pediatric multimodal protocols (7, 13). Treatment for adult Wilms tumor typically involves multimodal therapy, including surgery and chemotherapy, with radiotherapy used according to stage, histologic risk, and other clinical factors (14). Nephrectomy with complete tumor resection remains the cornerstone of local therapy, with particular attention to avoiding intraoperative tumor rupture or spill. Systematic sampling of regional lymph nodes at nephrectomy is important for accurate staging and risk-adapted postoperative treatment planning (15).

Pediatric Wilms tumor is commonly managed according to two major cooperative-group strategies: the SIOP protocol recommends preoperative chemotherapy to reduce tumor size, facilitate surgery, and potentially reduce the risk of intraoperative tumor rupture, whereas the COG approach emphasizes upfront nephrectomy, with surgery performed before chemotherapy (1, 13). A key advantage of upfront surgery is that it provides untreated tumor tissue for histopathologic evaluation and permits assessment of local extent and regional lymph node status, which informs postoperative risk stratification and adjuvant therapy. In adults, preoperative distinction between Wilms tumor and other renal neoplasms, particularly renal cell carcinoma, can be challenging. Accordingly, when renal cell carcinoma is the leading preoperative diagnosis and the tumor is resectable, upfront nephrectomy may be a reasonable approach; however, management should be individualized through multidisciplinary discussion. In line with this approach, our patient underwent nephrectomy, which achieved complete local resection without tumor rupture and with negative surgical margins. The pathologic findings, together with regional lymph node assessment and operative findings, informed postoperative staging and risk-adapted adjuvant treatment.

Chemotherapy is a cornerstone of Wilms tumor treatment and is associated with improved outcomes when integrated into risk-adapted multimodal therapy. Because adult-specific prospective protocols are lacking, treatment for adult Wilms tumor is generally adapted from pediatric cooperative-group strategies, including those developed by the Children's Oncology Group (COG) and the International Society of Paediatric Oncology (SIOP). These risk-adapted regimens commonly use vincristine and dactinomycin, with doxorubicin added for patients with higher-stage or higher-risk disease. In the COG/NWTS-based framework, EE-4A (vincristine plus dactinomycin) is an established postoperative regimen for patients with completely resected, favorable-histology stage I–II disease, whereas DD-4A, which adds doxorubicin, is generally used for favorable-histology stage III–IV disease and selected higher-risk clinical settings (15, 16). The roles of immunotherapy and targeted therapy in adult Wilms tumor remain investigational, and current evidence is limited primarily to small studies and case reports. Their use should therefore be considered on an individualized basis, preferably in the context of a clinical trial or multidisciplinary discussion (17, 18). In the present case, complete tumor resection was achieved without tumor rupture or spill, and the available regional lymph node assessment and staging evaluation showed no nodal or distant metastases. These findings were consistent with favorable-histology stage I Wilms tumor. On the basis of the final stage and favorable histology, EE-4A was selected as adjuvant therapy. This regimen avoided doxorubicin exposure and its associated cumulative cardiotoxicity risk. Postoperative radiotherapy was not administered because the tumor was completely resected and the final pathology showed favorable histology without indications for radiotherapy under the treatment framework used. A SIOP-based strategy represents an alternative pediatric treatment framework. SIOP protocols typically employ preoperative chemotherapy followed by nephrectomy, with postoperative treatment tailored to post-chemotherapy stage, histologic risk, and surgical findings. However, preoperative recognition of Wilms tumor in adults is challenging because renal cell carcinoma is often the leading diagnostic consideration for an adult renal mass. When the mass is resectable and renal cell carcinoma is the leading preoperative diagnosis, upfront nephrectomy may be reasonable; definitive histopathologic diagnosis and postoperative staging can then guide adjuvant treatment. Evidence supporting pediatric-style regimens in adults is limited primarily to retrospective series and case reports. Available reports support consideration of risk-adapted multimodal therapy, although the magnitude of benefit and the optimal regimen for adults remain uncertain. In the absence of prospective adult-specific trials, the use of EE-4A was adapted from pediatric risk-stratified practice and selected after multidisciplinary review. In pediatric-derived treatment frameworks, radiotherapy is generally considered for selected patients with higher-stage disease, adverse local surgical findings, or unfavorable histology to reduce the risk of locoregional recurrence (19).

During treatment, the patient was monitored for vincristine-related neurotoxicity, including peripheral neuropathy, constipation, and motor dysfunction, as well as actinomycin D-related hematologic and hepatic toxicity. No clinically significant treatment-related adverse events were documented during treatment or follow-up. The patient completed the planned chemotherapy regimen and resumed normal daily activities after treatment. When pediatric chemotherapy regimens are used in adults, toxicity may differ from that observed in pediatric populations. Close clinical, hematologic, and biochemical monitoring is therefore warranted.

Molecular testing was not performed in this patient because of financial constraints. This represents an important limitation, as the prognostic relevance of specific molecular alterations in Wilms tumor is increasingly recognized. In particular, chromosome 1q gain has been consistently associated with an increased risk of relapse and inferior survival. Combined loss of heterozygosity (LOH) at 1p and 16q is also associated with less favorable outcomes and has been incorporated into risk stratification in selected pediatric cooperative-group protocols. The absence of these analyses prevented a more refined molecular risk assessment and may have limited identification of molecular high-risk features that could have informed treatment intensity and surveillance planning. Given the patient's completely resected, favorable-histology stage I disease, EE-4A was selected on the basis of conventional clinicopathological risk factors. However, the absence of molecular profiling should be considered when interpreting prognosis, and molecular testing should be pursued whenever feasible, particularly when results may affect risk stratification or management.

Although this patient remained recurrence-free at 1 year, this duration of follow-up is insufficient to determine long-term oncologic outcome. Reported outcomes for adult Wilms tumor (AWT) have historically been less favorable than those for pediatric Wilms tumor, although comparisons are limited by differences in stage distribution, treatment era, and the quality of available evidence. A fundamental challenge is the absence of prospective, adult-specific treatment guidelines and trial data; treatment decisions are therefore generally adapted from pediatric cooperative-group protocols and informed by retrospective series and case reports. This limited evidence base makes it difficult to define the optimal treatment approach and to deliver consistent care across institutions. Pediatric-style, risk-adapted multimodal therapy is commonly used in adults; however, the extent to which it improves adult outcomes has not been established in prospective studies. Potential contributors to poorer reported adult outcomes include delayed or challenging diagnosis, more advanced stage at presentation, treatment heterogeneity, comorbidities, and possible differences in tumor biology. The contribution of unfavorable histology and reduced tolerance of intensive therapy in adults remains uncertain and should not be assumed without supporting data. Importantly, the absence of recurrence at 1 year should be interpreted cautiously. Wilms tumor relapse may occur beyond the first postoperative year, and the timing and pattern of recurrence in adults are not well defined because AWT is rare and most published reports have limited follow-up. Accordingly, continued long-term surveillance is warranted, and durable disease control cannot be concluded on the basis of 1-year follow-up alone. Continued efforts to improve diagnostic accuracy, validate adult-specific risk stratification, and develop prospective multicenter collaborative studies are needed to strengthen the evidence base and improve outcomes for this rare population.

## Data Availability

The original contributions presented in the study are included in the article/Supplementary Material, further inquiries can be directed to the corresponding author.

## References

[1] Bhutani N Kajal P Sharma U. Many faces of Wilms tumor: recent advances and future directions. Ann Med Surg (Lond). (2021) 64:102202 10.1016/j.amsu.2021.102202 33747498 PMC7970064

[2] Lachkar S Ibrahimi A Boualaoui I Sayegh HE Nouini Y. Adult nephroblastoma or Wilms’tumor: a rare entity—case report. Urol Case Rep. (2024) 57:102858. 10.1016/j.eucr.2024.102858 39435432 PMC11492433

[3] Alexandre AM Lori JM Mtaturu GF Sensa VP Nsato SB Mbezi MA. Adult Wilms tumor with inferior vena cava thrombus on an incomplete duplex collecting system ureter fissus proximalis managed at a tertiary hospital in Tanzania: a case report and literature review. Clin Case Rep. (2025) 13(1):e70136. 10.1002/ccr3.70136 39844890 PMC11752138

[4] Howell J Maughan B Fluchel M Poppe M. Pediatric regimens in the treatment of adult-onset, metastatic Wilms tumor: a case report. Urol Case Rep. (2024) 56:102799. 10.1016/j.eucr.2024.102799 39119470 PMC11305201

[5] Yantisetiasti A Sihombing AT Machbub HR. Adult Wilms tumor: a rare case report from tertiary hospital in Bandung, West Java, Indonesia. Urol Case Rep. (2025) 62:103142. 10.1016/j.eucr.2025.103142 40799252 PMC12341513

[6] He Y Yang F Gao Q Xiang Y Chen Z Chen X. Case series of adult Wilms’tumor and review of the literature. AME Case Reports. (2025) 9:39. 10.21037/acr-24-208 40330928 PMC12053429

[7] Balis F Green DM Armstrong A Aye J Benedetti D Brown B. Wilms tumor, version 2.2025, NCCN clinical practice guidelines in oncology. J Natl Compr Canc Netw. (2025) 23(8):319–42. 10.6004/jnccn.2025.0037 40763792

[8] Tripathi S Mishra A Popat VC Husain SA. Wilms’tumor in adults-conventional and unconventional presentations of a rare entity with a review of literature. J Kidney Cancer VHL. (2021) 8(2):40–8. 10.15586/jkcvhl.v8i2.186 34322362 PMC8297498

[9] Huszno J Starzyczny–Słota D Jaworska M Nowara E. Adult Wilms’tumor—diagnosis and current therapy. Cent European J Uro. (2013) 65:39–44. 10.5173/ceju.2013.01.art12 PMC3921847 24578986

[10] Huang J Liao Y Qiu M. Spontaneous rupture of adult Wilms’tumor: a case report and review of the literature. Can Urol Assoc J. (2015) 9(7–8):531. 10.5489/cuaj.2539 PMC4514509 26279733

[11] Szychot E Apps J Pritchard-Jones K. Wilms’tumor: biology, diagnosis and treatment. Transl Pediatr. (2014) 3(1):12–24. 10.3978/j.issn.2224-4336.2014.01.09 26835318 PMC4728859

[12] Modi S Woi Tiang K Inglis P Collins S. Adult Wilms’tumour: case report and review of literature. J Kidney Cancer VHL. (2016) 3(2):1–7. 10.15586/jkcvhl.2016.52 28326278 PMC5347375

[13] Pritchard-Jones K Bergeron C De Camargo B van den Heuvel-Eibrink MM Acha T Godzinski J. Omission of doxorubicin from the treatment of stage II-III, intermediate-risk Wilms’tumour (SIOP WT 2001): an open-label, non-inferiority, randomised controlled trial. Lancet (London, England). (2015) 386(9999):1156–64. 10.1016/S0140-6736(14)62395-3 26164096

[14] Fernández-Ferreira R Torres-Zazueta JM Martínez-Medrano C Meléndez-Mendoza A Tavares-García S Muñoz Rubiano MA. Nephroblastoma in older adult: case report and review of literature. Case Rep Oncol. (2024) 17(1):818–30. 10.1159/000540279 39144242 PMC11324259

[15] Sforza S Palmieri VE Raspollini MR Roviello G Mantovani A Basso U. Robotic approach with neoadjuvant chemotherapy in adult Wilms’tumor: a feasibility study report and a systematic review of the literature. Asian J Urol. (2023) 10(2):128–36. 10.1016/j.ajur.2021.10.004 36942112 PMC10023547

[16] Sakthivel V Ismail Z A Vijayabalan D. Recent improvements in adult Wilms tumor diagnosis and management: review of literature. J Kidney Cancer VHL. (2023) 10(3):32–6. 10.15586/jkcvhl.v10i3.281 37583880 PMC10423726

[17] Zhao Q Xiong Q Song Q. Metastatic adult Wilms’tumor managed by chemotherapy, immunotherapy and target therapy: a case report. Future Sci OA. (2024) 10(1):FSO915. 10.2144/fsoa-2023-0156 38817367 PMC11137782

[18] Kroll MR Au C Slostad J Christ TN Papas SG Tan A. Case report: metastatic BRAF V600E-mutated adult Wilms’tumor with robust response to BRAF/MEK inhibitor therapy. Front Oncol. (2024) 14:1376270. 10.3389/fonc.2024.1376270 39234402 PMC11373342

[19] Sudour-Bonnange H Coulomb-Lherminé A Fantoni JC Escande A Brisse HJ Thebaud E. Standard of care for adult Wilms tumor? From adult urologist to pediatric oncologist. A retrospective review. Bull Cancer. (2021) 108(2):177–86. 10.1016/j.bulcan.2020.09.007 33129487

