# Supplementary material for: Adult Wilms tumor revealed after radical nephrectomy for a renal mass initially suspected to be clear-cell renal cell carcinoma: a case report
Source: Front Surg. 2026 Sep 3;13:1889237. doi: 10.3389/fsurg.2026.1889237 (PMC13582410; doi:10.3389/fsurg.2026.1889237)
Supplement: Supplementary file 1 [file Table1.docx]

## **Patient Clinical Timeline**

| **Date/Period** | **Clinical events, investigations, interventions, and outcomes** |
| --- | --- |
| ****Mid-to-late December 2023 (approximately)**** | A 39-year-old man presented with a 2-week history of progressively worsening, persistent dull pain in the right lower abdomen. The pain was nonradiating and had no clear association with posture or food intake. He denied weight loss, fatigue, fever, night sweats, gross hematuria, nausea, or vomiting. |
| ****January 2, 2024**** | The patient was referred to the Department of Urology because of persistent, progressively worsening right lower abdominal pain. His vital signs were within normal limits. Physical examination revealed mild deep tenderness in the right lower abdomen, with no palpable abdominal mass, rebound tenderness, or guarding. There was no bilateral costovertebral angle tenderness. His past medical history, surgical history, trauma history, social history, and family history were unremarkable. |
| ****January 2024: preoperative laboratory evaluation**** | Complete blood count, liver and renal function tests, and serum electrolyte levels were within normal limits. Urinalysis showed no hematuria or proteinuria.. |
| ****January 2024: preoperative imaging evaluation**** | Chest radiography showed no evidence of pulmonary metastases. Contrast-enhanced computed tomography (CT) of the abdomen and pelvis demonstrated a well-circumscribed, heterogeneous mass arising from the upper pole of the right kidney, measuring approximately 7.2 × 6.5 × 6.6 cm. The lesion contained nodular hyperattenuating foci and patchy mildly hypoattenuating areas, with delayed heterogeneous enhancement. No retroperitoneal lymphadenopathy or abdominal or pelvic metastases were identified on CT. |
| ****January 11, 2024**** | The patient underwent retroperitoneal laparoscopic radical nephrectomy, with removal of the renal tumor and regional lymph nodes. The renal artery and vein were identified, ligated, and divided. The surgical specimen was removed intact, without tumor rupture or spillage, and no intraoperative complications were reported. Gross examination revealed a gray-white to gray-brown, solid tumor measuring approximately 5.0 × 5.0 × 3.0 cm, located in the mid-to-upper pole of the kidney. The tumor involved the renal parenchyma and pelvicalyceal system but was confined within the renal capsule, with no gross extension into the perinephric tissue. All examined surgical margins were negative for tumor. |
| ****January 16, 2024**** | Histopathological examination confirmed an epithelial-predominant Wilms tumor (nephroblastoma). Microscopically, the tumor was composed of primitive epithelial cells with a high nuclear-to-cytoplasmic ratio, arranged in trabecular patterns and rosette-like tubular structures, with brisk mitotic activity. Immunohistochemical staining was positive for WT1, cytokeratin, vimentin, and PAX8, with a Ki-67 labeling index of approximately 60%. CD117 showed focal weak positivity. CK7, CR, P504S, inhibin, and CD10 were negative. All five examined hilar lymph nodes were negative for metastasis. No lymph nodes were identified in the examined perirenal adipose tissue. |
| ****Mid-to-late January 2024: postoperative staging and multidisciplinary assessment**** | Based on the pathological and radiological findings, the tumor was classified as stage I Wilms tumor, with complete resection, negative regional lymph nodes, and no radiological evidence of distant metastasis. Following multidisciplinary discussion involving urology, medical oncology, radiology, and pathology, the patient was considered to have low-risk disease according to a COG-based risk-stratification approach. Molecular analyses, including assessment of 1q gain, 11p15 loss of heterozygosity, and combined 1p and 16q loss of heterozygosity, were not performed. |
| ****February 4, 2024**** | Adjuvant chemotherapy based on the Children's Oncology Group (COG) EE-4A regimen was initiated. The regimen consisted of vincristine (1.5 mg/m² intravenously, with the applicable maximum dose specified) and dactinomycin (actinomycin D; dose and schedule according to the institutional EE-4A protocol). Complete blood counts, liver and renal function tests, clinical status, and interval imaging studies were monitored throughout treatment. |
| ****February 4–June 15, 2024**** | The patient ****completed the planned course of adjuvant chemotherapy based on the EE-4A regimen****. No clearly documented severe treatment-related adverse events were reported in the available clinical records. |
| ****June 2025 (approximately 1 year after completion of multimodal treatment)**** | The patient remained asymptomatic and maintained a good functional status. Follow-up contrast-enhanced CT of the chest, abdomen, and pelvis showed no evidence of residual or recurrent tumor, regional lymph-node metastasis, or distant metastasis. The patient had no evidence of disease at the most recent follow-up and remained under regular clinical and radiological surveillance. |
